# Two institutes’ experience in laparoendoscopic “rendezvous” technique for patients undergoing laparoscopic cholecystectomy for stones in the gallbladder and bile duct: a prospective randomized comparative clinical trial

**DOI:** 10.1007/s13304-024-01973-6

**Published:** 2024-09-25

**Authors:** Mohamed Farid, Azza Baz, Alaaedin Ramadan, Mohamed Elhorbity, Ashraf Amer, Ahmed Arafa

**Affiliations:** 1 Assistant Professor of General Surgery, Zagazig University, Zagazig City, Egypt; 2 Associate Fellow of General Surgery, Al-Ahrar Teaching Hospital, Zagazig City, Egypt; 3 Lecturer of General Surgery, Zagazig University, Zagazig City, Egypt; 4 Assistant Professor of General Surgery, Banha University, Banha City, Egypt; 5 Fellow of General Surgery, Al-Ahrar Teaching Hospital, Zagazig City, Egypt

**Keywords:** Laparoscopic cholecystectomy (LC), Endoscopic sphincterotomy (ES), Common bile duct stones (CBDS), Laparoendoscopic rendezvous (LERV), Intraoperative endoscopic retrograde cholangiopancreatography (IO-ERCP)

## Abstract

There is still disagreement on the best treatment option for cholecystocholedocholithiasis. Although there are some benefits to the single-step procedure, the “laparoendoscopic rendezvous” (LERV) technique that include a lower risk of post-ERCP pancreatitis and a shorter hospital stay, the standard technique is still the two-step approach for clearing the common bile duct (CBD) using ERCP and then performing a laparoscopic cholecystectomy. The purpose of this study was to assess the effectiveness and safety of the LERV technique vs. the standard two-step approach. Four hundred thirty-six patients with symptomatized concomitant stones at both the gall bladder (GB) and the (CBD), at two gastroenterology centers in Zagazig city, Egypt, from January 2010 till April 2022, were analyzed. Patients were randomly divided into two equally groups. The overall length of hospital stay was the primary outcome, and the success of CBD clearance and morbidity, particularly post-ERCP pancreatitis, were the secondary endpoints. The LERV group experienced a significantly shorter hospital stay (median 2(2–8) days compared to 4.5 (4–11) days for the two-stage approach (*p* < 0.001)). The two groups did not differ in terms of CBD clearing success. Also, there was no significant difference in the number of patients with post-ERCP pancreatitis between the LERV group [14 patients (6.4%)] and the two-stage approach [26 patients (11.9%)] with *p* value = 0.703. For patients with cholecystocholedocholithiasis, the optimal treatment must be determined by the knowledge and resources that are accessible locally. Our data further supported the idea that treating patients with cholecystocholedocholithiasis in one stage is a safe and successful strategy.

## Introduction

Concomitant stones in the common bile duct (CBD) are found in up to 16% of patients with gallbladder stones [1]. The occurrence of stones in both the gallbladder and the common bile duct is known as cholecystocholedocholithiasis. The vast majority of persons with gallbladder stones (cholelithiasis) are unaware of their presence, and up to 25% of initially asymptomatic patients will develop biliary colic over a 10-year period of follow-up [2].

Preoperative symptoms or signs of jaundice, pancreatitis, or cholangitis, abnormalities in liver function tests, or imaging revealing duct widening or actual ductal stones can all be indicators of CBD stones. Up to 25% of CBD stones will be discovered unexpectedly during surgery [3]. According to several studies, a combination of increased bilirubin and alkaline phosphatase levels increases the chance of CBDS by over 70% [4].

CBDS have an unknown natural history, but complications appear to be more frequent and severe than asymptomatic gallstones [5]. The National Institutes of Health consensus argues that the best therapy involves detecting them and removing them either before a planned laparoscopic cholecystectomy, during the cholecystectomy, or thereafter [6].

Despite significant advancements over the past 10 years and the fact that laparoscopic cholecystectomy (LC) has become the accepted treatment for gallbladder stones, there is still disagreement over the optimal management of CBD stones [7]. Patients with gallstones and CBDS were treated with open cholecystectomy and common bile duct exploration prior to the advent of endoscopy and laparoscopy [8]. A multidisciplinary strategy combining ES and LC, in particular, as a two-stage, sequential treatment and, more recently, in a single operating session have been widely used, since the inception of laparoscopic surgery [9]. Treatment in two stages, which combines a preoperative ES followed by LC (sequential treatment), combines the morbidity of the two surgeries [10].

For the performance of the two procedures in a single session, this combination approach has been used to improve patient compliance and shorten hospital stays. The intraoperative ERCP has been performed immediately before LC [11], during LC, as a rendezvous technique, in which a guidewire or a Dormia basket is passed through the cystic duct into the duodenum and grasped with the duodenoscopy, allowing the papilla to be cannulated more easily. The last option approach will avoid unnecessary cannulation of the pancreatic duct and accordingly most likely reduce the risk of pancreatitis [12].

The novel advancement of perioperative ES, which is performed immediately after LC (one-session treatment) and under the same general anesthesia, looks to be a fascinating option for CBDS treatment that is minimally invasive [13]. In addition, the treatment of CBDS during LC using an intraoperative method of ERCP in a single-step treatment which aids the patient by reducing the therapy from a two-step surgery to a single-step operation under general anesthesia. It reduces the chance of pancreatitis induction, avoids two further interventions, and avoids CBD exploration [14].

We aimed in this study to evaluate the one-stage LC with intraoperative endoscopic sphincterotomy (IOES) vs. two-stage preoperative endoscopic sphincterotomy (POES) followed by LC for the treatment of cholecystocholedocholithiasis.

## Patients and methods

### Study design

This is a two-center prospective randomized comparative clinical trial study that was completed for 436 patients at 2 gastroenterology centers in Zagazig city; 264 patients at the Gastrointestinal Surgery Unit in the Zagazig University Hospitals and 172 patients at gastroenterology unit et al.-Ahrar teaching hospital from January 2010 till April 2022.

### Study approval

Ethical approval for this study was granted by the Institutional Review Board of the Zagazig University, with an IRB registration number ZU-IRB#10,129/20–11-2022. Also, this clinical trial was registered at the ClinicalTrials.gov with an Identifier: NCT05734144. The work has been reported in line with Consolidated Standards of Reporting Trials (CONSORT) Guidelines [15].

### Participants

#### The inclusion criteria

The study included patients having stone in the gallbladder and concurrent CBDS, as determined by MRCP or US. Patients with acute cholecystitis, acute cholangitis, obstructive jaundice, and those with highly suspicious criteria for CBDS, such as dilated CBD on US examination > 7 mm in diameter without obvious CBDS, high serum bilirubin level, and/or high serum alkaline phosphatase level, were also included in this study.

#### Exclusion criteria

The study excluded patients with (1) a history of hepatobiliary surgery as choledochoduodenal anastomosis, (2) a previous ERCP attempt, (3) a previous upper abdominal surgery as total or partial gastric resection, (4) morbid obesity, (5) uncorrectable coagulopathy, (6) age < 18 years or > 80 year or (7) American Society of Anesthesiology (ASA) class 4 and 5 disease, as well as (8) patients who refused to give consent.

### Workup and randomization of patients

**Fig. 1:**
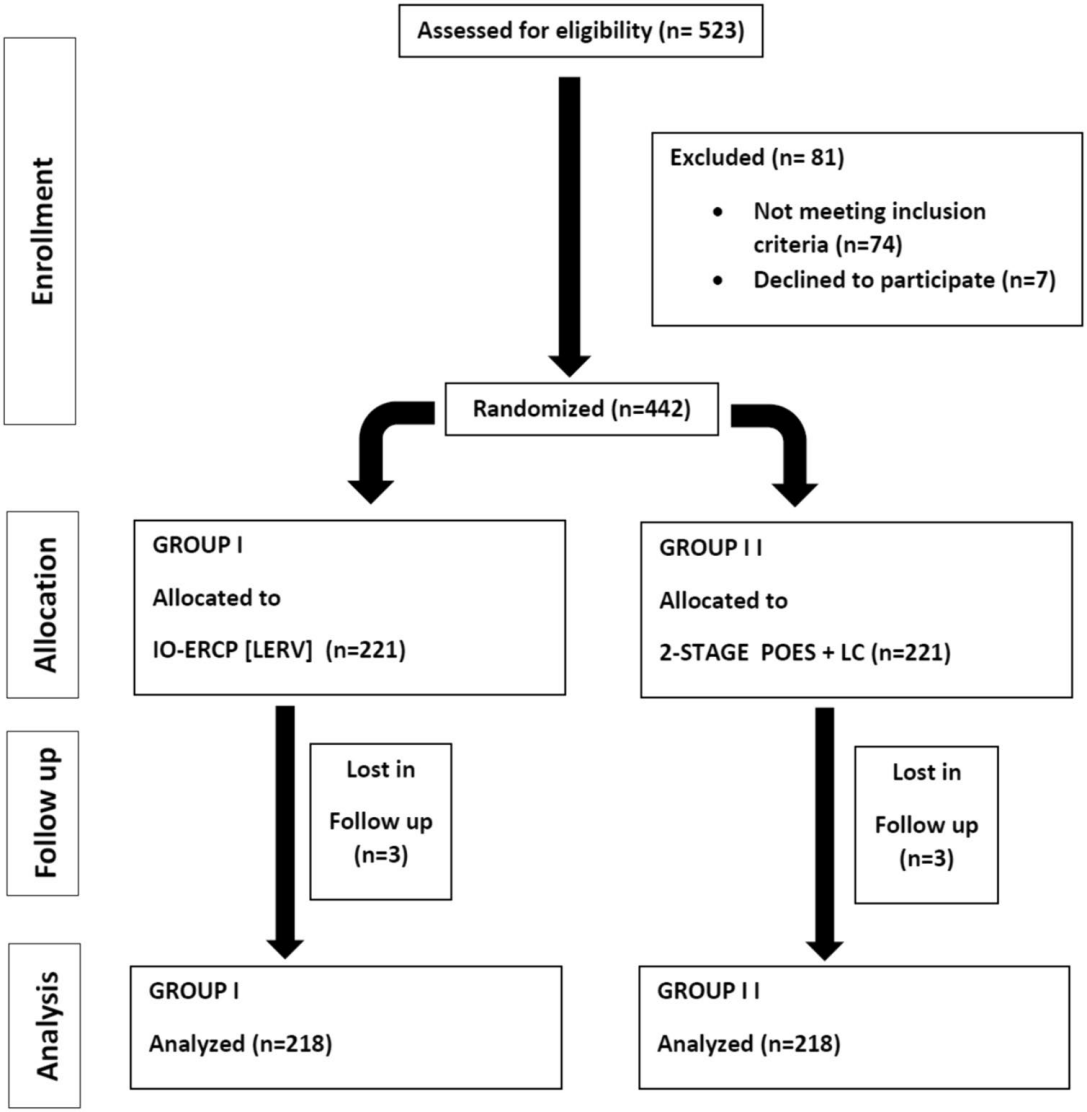
Flow chart for the allocation process throughout the trial

From January 2010 till April 2022 a total number of 523 patients with both gall bladder stones and with suspected or confirmed CBDS were enrolled in this study. Eighty-one patients were excluded either due to refusal to be included, incomplete data or did not complete both steps within our centers. So, a final 442 patients were randomly allocated and divided into 2 equal groups, the first group (*n* = 221) was treated by a single-step procedure combining LC and IOES while the second (control) group (*n* = 221) was treated by two-stage (sequential treatment) POES followed by LC. Scheduling for laparoscopic cholecystectomy in the control group ranged from within 8 weeks after preoperative ERCP. Six patients were lost in follow-up, so the final number of patients analyzed in this study was four hundred thirty-six patients (*n* = 218 for each group). The patients were randomized into two equal groups, utilizing computer-generated random numbers in sealed envelopes numbered serially. See the flow chart for the allocation process throughout the trial (Fig. 1).

Patients were assessed clinically and subjected to standard hematologic and biochemical assays, as well as imaging. A magnetic resonance cholangiopancreatography (MRCP) was performed to confirm the presence of stones in the CBD if a trans-abdominal ultrasound examination revealed gallbladder stones and suspicion of CBD stones with a CBD diameter greater than 7 mm before the patient’s inclusion in the study and randomization. The demographic, clinical, and biochemical information of the patients, as well as their radiologic parameters, were all documented.

## The procedures

### The laparoendoscopic rendezvous (LERV) technique

#### The main principles of LERV technique consists of

1. An antegrade transcystic cannulation of the bile duct during laparoscopic cholecystectomy, with a guidewire that can be retrieved with a duodenoscope, thus facilitating retrograde bile duct cannulation.
2. An over-the-wire sphincterotome is then inserted and standard maneuvers of endoscopic common bile duct stones clearance are performed.
3. The procedure is then completed by cholecystectomy in one procedure.

#### The steps of LERV technique are

**Fig. 2:**
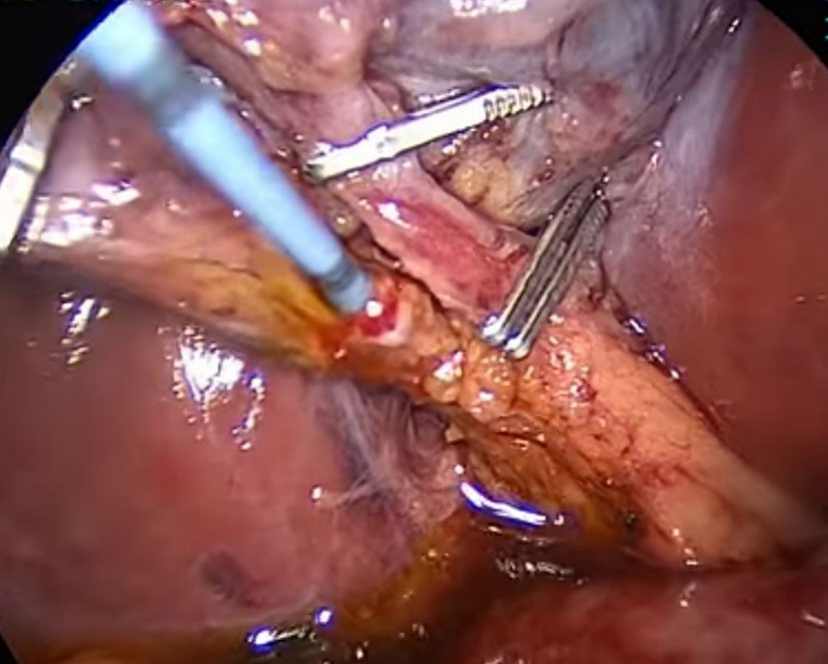
A cholangio-catheter used to catheterize the cystic duct

**Fig. 3:**
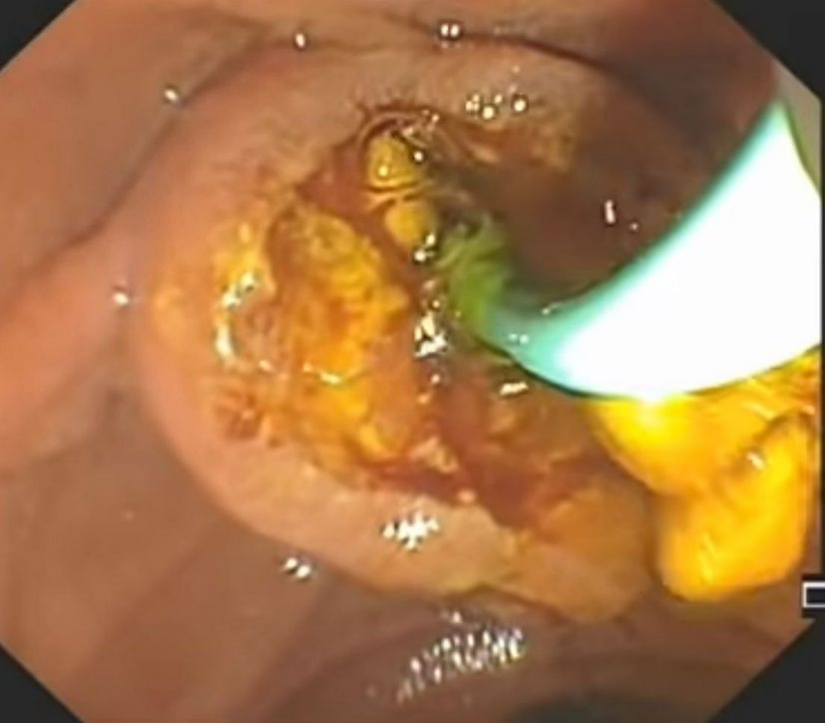
Extraction of stones from CBD by Fogarty balloon

According to usual procedure, a laparoscopic cholecystectomy was performed with the standard four trocars technique. To avoid intestinal distension with air during ERCP, LC was started first. The cystic artery was located, clipped, and cut after the Calot’s triangle was dissected. The cystic duct was clipped high toward the GB, and a tiny incision was made near the clip for introduction of the cholangio-catheter to confirm the presence of CBD stones. A cholangio-catheter (4 Fr. Ureteric catheter) was used to catheterize the cystic duct (Fig. 2), and IOC was obtained following injection of 10 cc of diluted urografin utilizing a C-arm X-ray. The decision to perform IO-ERCP was made when the IOC revealed the presence of a CBDS or when the anatomy of the CBD was suspicious for the presence of a CBDS. A 0.035-inch guidewire was inserted into the cystic duct and progressed down past the sphincter of Oddi and into the duodenum by the surgeon. The duodenoscope was placed by the surgeon with the patient remaining in the supine position and advanced to the second portion of the duodenum, where the guidewire was encountered upon detection of the papilla (rendezvous technique) and was used to guide cannulation of the CBD using a sphincterotome. Endoscopic retrograde cholangiography (ERCP) was performed using diluted urografin given through the sphincterotome. A sphincterotomy was performed when a CBDS was discovered. The CBDS was subsequently removed using a retrieval Fogarty balloon (8.5 or 11.5 mm) (Fig. 3). After that, a completion cholangiography was performed to ensure that the CBD was free of stones. Care was taken to remove all gas from the stomach at the end of each ERCP to make the LC easier to complete.

### Preoperative endoscopic sphincterotomy (POES) followed by LC

The ERCP procedure was performed as an outpatient treatment. On the morning of the procedure, all of the patients were fasting. The procedure was carried out while the patient was sedated with intravenous medication. A side-viewing duodenoscope was used for the ERCP operation, which was introduced trans-orally until the second portion of the duodenum. A wire-guided sphincterotome and a hydrophilic guidewire were used to selectively cannulate the bile duct. A contrast dye was administered after guidewire-assisted cannulation to establish the presence of CBD stones. A biliary sphincterotomy was performed with a mix of cutting and coagulation current to extract the stones. To ensure that the bile duct was completely clear, a check cholangiogram was conducted. After the surgery, the patients were kept under monitoring for 6–8 h. Perforation, hemorrhage, pancreatitis, or cholangitis were all noted as consequences. From the day before the treatment to 5 days afterward, the patients were given pre-procedure oral broad-spectrum antibiotics. Median time interval for LC, in PO-ERCP group, was 7 weeks with a range (6–8) weeks after endoscopic extraction of the CBD stones.

## Follow-up assessment

Because of the likelihood of postoperative biliary illness, patients were instructed to report any clinical symptoms or signs, as well as any laboratory or imaging data, following discharge. Patients were followed up on at 1 week, 6 weeks, 3 months, 6 months, and up to 1 year after surgery, or at any time if symptoms appeared. The presence and severity of pain, wound state, history of jaundice, and any other issues were all documented. At a 6-week follow-up evaluation, overall satisfaction was assessed on a verbal rating scale with scores of 0 (not satisfied), 1 (partially satisfied), 2 (satisfied), or 3 (very satisfied). A 3-month follow-up evaluation included a trans-abdominal ultrasound and liver function tests to assess the CBD’s status.

## Statistical analysis

Continuous variables were expressed as the mean ± SD and median (range), and the categorical variables were expressed as a number (percentage). Continuous variables were checked for normality using Shapiro–Wilk test. Independent samples Student’s *t* test was used to compare two groups if normally distributed variables, while Mann Whitney U test was used for non-normally distributed variables. Percent of categorical variables were compared using Pearson’s Chi-square test or Fisher’s exact test when was appropriate. All tests were two tailed. *p* value < 0.05 was considered statistically significant. All statistics were performed using SPSS version 22.0 for windows (IBM Corp., Armonk, NY, USA).

## Results

Participant demographics and their findings in US examination are detailed in Table 1. History of biliary colic was found in 215 patients in IO-ERCP group vs. 216 patients in PO-ERCP group (*p* value = 1). History of preoperative jaundice was found in 96 patients in IO-ERCP group vs. 82 patients in PO-ERCP group. Thirty-four patients in IO-ERCP group had history of cholangitis vs. fifty patients in PO-ERCP group. Median time interval since ERCP in PO-ERCP group was 7 weeks with a range (6–8) weeks. Preoperative biliary pancreatitis was found in 28 in IO-ERCP group vs. 38 patients in PO-ERCP group. Ultrasound findings showed dilated CBD in 224 patients, ectatic CBD (around 7 mm in diameter) in 172 patients, normal CBD diameter in 40 patients. The CBD stones’ diameter ranged from tiny gravels to 13 mm.

**Table 1:** Patients characteristics and ultrasound findings among the studied groups

Patients’ characteristics	IO-ERCP group (*N* = 218)		PO-ERCP group (*N* = 218)		Test	*p* value
	No	%	No	%		
**Sex**						
Male	125	55.9%	62	28.4%	2.857a	0.091
Female	93	44.1%	156	71.6%		
**Age (years)**						
Mean ± SD	39.56 ± 12.31		44.44 ± 10.18		− 1.298b	0.203
Median (range)	37.50 (25 – 60)		42.50 (18–65)			
**Biliary colic**						
Absent	3	1.4%	2	0.9%	0.000a	1.000
Present	215	98.6%	216	99.1%		
**History of preoperative jaundice**						
Absent	122	55.9%	136	62.4%	0.114a	0.735
Present	96	44.1%	82	37.6%		
**History of cholangitis**						
Absent	182	83.5%	168	77.1%	0.177	1.000
Present	34	16.5%	50	22.9%		
**Time interval since ERCP (weeks)**						
Mean ± SD			7.11 ± 0.83			
**Biliary pancreatitis**						
Absent	190	87.2%	180	82.6%	0.232a	1.000
Present	28	12.8%	38	17.4%		
**Ultrasound findings**						
Dilated CBD	94	43.1%	130	59.6%	1.950a	0.377
Ectatic CBD	108	49.5%	64	29.4%		
Normal CBD diameter	16	7.4%	24	11.0%		

Categorical variables were expressed as number (percentage); continuous variables were expressed as mean ± SD and median (range); a: Chi-square test; b: independent samples Student’s *t* test; *p* value < 0.05 is significant

Patients with normal CBD on US examination had history of pancreatitis, preoperative jaundice, and/or elevated serum bilirubin or alkaline phosphatase denoting suspicion for the presence of CBDS. Magnetic resonance cholangiography (MRCP) was performed and showed the presence of a tiny stone at the terminal end of the CBD in 36 patients with pancreatitis who had a normal CBD on US examination.

Patients’ operative data and outcomes were detailed in Table 2. Operation duration range was from 50 to 90 min in IO-ERCP group vs. (40–70) min in PO-ERCP group with *p* value < 0.001.

**Table 2:** Patients’ operative data and outcomes among the studied groups

	IO-ERCP group (*N* = 218)		PO-ERCP group (*N* = 218)		Test	*p* value
	No	%	No	%		
Yes	204	93.6%	180	82.6%	1.125a	0.603
Suspected	14	6.4%	38	17.4%		
Single CBD stone	109	50.0%	132	60.6%	0.450a	0.502
Multiple CBD stones	109	50.0%	86	39.4%		
Mean ± SD	78.17 ± 12.72		57.44 ± 8.80		− 4.038c	< 0.001
Absent	204	93.6%	190	88.9%	0.364a	1.000
Present	14	6.4%	28	11.1%		
No	1	0.5%	3	1.4%	2.118a	0.886
Yes	217	99.5%	215	98.6%		
Absent	204	93.6%	192	88.1%	1.125a	0.703
Present	14	6.4%	26	11.9%		
Mean ± SD	2.94 ± 1.55		5.94 ± 2.79		− 4.196c	< 0.001
Median (range)	2 (2–8)		4.50 (4–11)			
No	25	11.5%	36	16.5%	0.232a	1.000
Yes	193	88.5%	182	83.5%		

Categorical variables were expressed as number (percentage); continuous variables were expressed as mean ± SD and median (range); a: Chi-square test; c: Mann–Whitney U test; *p* value < 0.05 is significant

The postoperative course was smooth in all patients. The range of postoperative hospital stay was (2–8) days in the first group vs. (4 – 11) days in the second group with a statistically significant short hospital stay in the first group (*p* value < 0.001). Return of peristaltic activity was noted on the same evening with passage of flatus on the next day with immediate start of feeding in all the 436 patients who had their cholecystectomy completed laparoscopically. No statistically significant difference was observed between the two groups in CBDS clearance rate as the successful CBDS clearance rate was 99.5% in IO-ERCP group vs. 98.6% in PO-ERCP group with *p* value = 0.886. Although the postoperative pancreatitis occurred in 26 patients in the second group vs. 14 patients in the first group, this low incidence of postoperative pancreatitis in the first group was not statistically significant (*p* value = 0.703). Minimal postoperative complications were noted in the form of mild basal lung atelectasis, mild fever, and mild postoperative pain in 180 patients. These complications were minor and did not require special interference, and patients were discharged in good health. Patient satisfaction was 88.5% in IO-ERCP group vs. 83.5% in PO-ERCP group.

## Discussion

When CBD stones coexist with gallbladder stones, surgeons now attempt to avoid open duct exploration and have a variety of therapeutic alternatives. So, numerous studies have been published on the effectiveness and safety of laparoscopic common bile duct exploration (LCBDE) [16] and the utility of endoscopic sphincterotomy (ES) before, during, and after laparoscopic cholecystectomy (LC) [17].

Before, the conventional treatment for CBDS was an open cholecystectomy, IOC, and then CBD exploration if stones were found. ERCP became a management option after the technological advancement in accurately detecting CBD stones preoperatively with CT scan or magnetic resonance cholangiopancreatography (MRCP). Then a sequential treatment strategy combining a preoperative ES with laparoscopic cholecystectomy (LC) was used since the beginning of laparoscopic surgery [18].

Timing of the LC after the ERCP is another commonly debated decision-making process in this two-phase procedure. Recommendations in the literature range from 24 to 72 h to 6 weeks, and this delay puts these patients at a 10% risk of recurrent CBD stones [19]. Drawbacks include longer hospital stays and expenditures (often two), the requirement for two anesthetic inductions, loss of compliance, and a higher conversion rate for LC (20%) [20]. One-stage, an intraoperative endoscopic sphincterotomy (IOES), has been proposed as laparoscopic and endoscopic skill grows. The choice is based on the surgeon’s preference as well as the availability of experienced endoscopists ready to provide such therapy as part of a multidisciplinary approach [21].

The new and practical one-stage procedure known as laparoendoscopic rendezvous (LERV) combines laparoscopic and endoscopic methods to remove gallbladder and common bile duct stones. Because a guidewire is put via the cystic duct, making it easier to identify and cannulate the papilla, the treatment also prevents cannulation of the pancreas and the development of a false channel [22].

Tables 3 and 4 display the features of the one-step procedure (LERV) vs. the standard two-step procedure in the studies that were included in this study discussion [23–28]. All of these studies and our study showed similar CBD clearance rate in both procedures with noticed more success clearance rate with one-step method.

**Table 3:** Characteristics of comparative studies for LERV, one-stage treatment, (LC + IO ES)

Study	Country + year	Sample size	Age	Total Operative time (min)	CBD clearance rate (*n*)	Overall morbidity (*n*)	Hospital stay (d)
El-Geidie et al. [23]	Egypt 2011	98	31.2 (20–67)	112 (60–180)	96/98	4/98	1.3 (1–4)
Tzovaras et al. [24]	Greece 2012	50	66 (22–87)	95 (65–200)	47/50	7/50	4 (2–19)
Gonzalez et al. [25]	Cuba 2016	99	58.4 (23 -87)	94.2 (45–300)	97.8%	0.00%	1.2
Liu et al. [26]	China 2017	32	42 ± 5.2	129 ± 16.3	31/32	17/32	7.5 ± 1.7
Garbarini et al. [27]	Italy 2017	143	59 (16–88)	215 (75–463)	142/143	15/143	7 (2–60)
Qian et al. [28]	China 2020	123	56.3 ± 15.5	60.5 ± 16.2	120/123	4/123	12 (3–20)
Present study	Egypt	218	37.50 (25 –60)	78.17 ± 12.72	217/218	14/2018	2 (2 – 8)

**Table 4:** Characteristics of comparative studies for preoperative ERCP + laparoscopic cholecystectomy (sequential treatment), (preop ES + LC)

Study	Country + year	Sample size	Age	Total Operative time (min)	CBD clearance rate (*n*)	Overall morbidity rate (*n*)	Hospital stay (*d*)
El-Geidie et al. [23]	Egypt 2011	100	27.5 (19–64)	98.4 (45–150)	95/100	6/100	3 (2–11)
Tzovaras et al. [24]	Greece 2012	49	69 (25–85)	79 (40–180)	44/49	7/50	5.5 (3–22)
Gonzalez et al. [25]	Cuba 2016	101	57.7 (20 -84)	98 (30–240)	93.3%	13.33%	3.1
Liu et al. [26]	China 2017	31	40 ± 6.1	151.3 ± 15.2	30/31	25/32	10.6 ± 2.5
Garbarini et al. [27]	Italy 2017	106	68 (23–88)	145 (30–397)	93/106	26/106	11 (3–40)
Qian et al. [28]	China 2020	137	58.2 ± 16.0	41.4 ± 20.7	5/137	14/137	18 (5–31)
Present study	Egypt	218	42.50 (18 – 65)	57.44 ± 8.80	215/218	36/2018	4.50 (4 – 11)

As regarding to the mean operative time while Gonzalez [25] and Liu [26], showed statistically significant shorter operative time during the one-step procedure, El-Geidie [23], Tzovaras [24], Garbarini [27], Qian [28], and the present study showed that the shorter operative time was statistically significant and documented for the two-step procedure. Present study also showed that the overall morbidity of the one-step procedure is lesser than the two-step procedure, but not statistically significant. Same results are also reported with the mentioned literatures [23–28].

Regarding the mean hospital stay, the present study reported statistically significant decrease of the total hospital stay results with the one-step procedure, which was coincidently similar to other literatures [23–28]. Liao et al., stated in a recent meta-analysis study of randomized trials for patients with cholecystocholedocholithiasis that the intraoperative-ERCP + LC (LERV) proved to be a safer course of treatment. It reduces postoperative complications, particularly postoperative pancreatitis, and shortens hospital stays [29]. This coincides with the findings and conclusion of the current study.

## Conclusion

According to the present study, we draw the conclusion that, in centers that can offer a team approach to the care of cholecystocholedocholithiasis, LC and intraoperative ERCP is an appealing option. For any patient with cholecystocholedocholithiasis, the optimal course of treatment must be determined by the knowledge and resources that are accessible locally. Finally, our data further supported the idea that treating patients with cholecystocholedocholithiasis in one stage is a safe and successful strategy. It may result in a lower incidence of overall morbidity and a shorter median length of hospital stay when compared to standard two-stage procedures.

## Declarations

### Data availability

All data generated or analyzed during this study are included in this article. Further enquiries can be directed to the corresponding author.

### Registry and the registration no. of the study/Ttial

The ClinicalTrials.gov with an Identifier: NCT05734144.

### Animal studies

N/A.
